# Electronic Cigarettes and Smoking Cessation in the Perioperative Period of Cardiothoracic Surgery: Views of Australian Clinicians

**DOI:** 10.3390/ijerph15112481

**Published:** 2018-11-07

**Authors:** Nia A. Luxton, Patti Shih, Muhammad Aziz Rahman

**Affiliations:** 1 Department of Psychology, Macquarie University, Sydney 2109, Australia; 2 Australian Institute of Health Innovation, Macquarie University, Sydney 2109, Australia; Patti.Shih@mq.edu.au; 3 Austin Clinical School of Nursing, La Trobe University, Melbourne 3084, Australia; m.rahman2@latrobe.edu.au

**Keywords:** tobacco, preoperative, surgery, electronic cigarette, surgeons, anaesthetists, nurses, physiotherapists

## Abstract

For patients who smoke, electronic cigarettes may offer a pathway to achieve tobacco abstinence and reduce the risk of postoperative complications. Clinicians have a pivotal role in supporting smoking cessation by patients with lung cancer and coronary artery disease throughout the perioperative period of cardiothoracic surgery. However, the views of Australian cardiothoracic clinicians on electronic cigarettes and smoking cessation are unknown. Semi-structured interviews were conducted with 52 cardiothoracic surgeons, anaesthetists, nurses and physiotherapists in six hospitals in Sydney and thematically analysed. Clinicians’ knowledge about electronic cigarettes and the regulatory environment surrounding them was limited. Clinicians believed that: electronic cigarettes, though unlikely to be safe, were safer than tobacco cigarettes; electronic cigarettes may have a harm reduction role in public health; and electronic cigarettes were a potential smoking cessation tool for the extraordinary circumstances of surgery. The professional role of a clinician and their views about electronic cigarettes as a perioperative smoking cessation aid had an influence on future clinician-patient interactions. Electronic cigarette use is increasing in Australia and clinicians are likely to receive more frequent questions about electronic cigarettes as a cessation aid. Stronger guidance for clinicians is needed on the topic of electronic cigarettes and cardiothoracic surgery.

## 1. Introduction

Clinicians play a pivotal role in supporting smoking cessation by patients with lung cancer and coronary artery disease undergoing cardiothoracic surgery. Continued tobacco smoking increases the risk of pulmonary and surgical complications, occurrence or re-occurrence of their primary and secondary disease, and death [1,2,3]. Numerous international and Australian best practice guidelines recommend that all clinicians assess smoking status and offer advice and support to enhance a patient’s motivation and cessation, irrespective of a patient’s desire to quit [4,5,6]. Yet the provision of perioperative cessation support by clinicians is negatively affected by factors such as the lack of hospital onsite cessation staff and resources, clinicians’ inadequate knowledge of available cessation services, and patients’ prior failed quit attempts with cessation pharmacotherapy [7,8]. The need for patients with coronary artery disease and lung cancer to explore different and novel methods to reduce or eliminate their tobacco use, has given rise to an increased number of patient-clinician discussions about electronic cigarettes [9,10,11]. This has led to a similar rise in studies exploring how clinicians involved in the care of patients with such tobacco-induced diseases, interact with their patients around this topic [12,13,14,15,16,17,18].

Electronic cigarettes are devices designed to deliver an aerosol by heating an e-liquid solution which contains optional flavouring, additives propylene glycol, and vegetable glycerine, and is available with or without nicotine [19]. There is an ongoing worldwide public health debate about the impacts of electronic cigarettes. Those against electronic cigarettes consider them to be potentially harmful devices that will renormalise smoking and provide a gateway to smoking specifically amongst young non-smokers [20,21,22,23]. Those for electronic cigarettes view them as a method of tobacco harm reduction, as they do not produce the dangerous combustion by-products of conventional tobacco cigarettes and may help people reduce or quit tobacco when other cessation attempts have failed [24,25]. Indeed, the National Institute for Health Care and Excellence (NICE) guidance in the United Kingdom has endorsed electronic cigarettes as a method of harm reduction, and advises clinicians engaging with patients who smoke to include electronic cigarettes in the discussion of nicotine replacement therapy (NRT) [26]. Electronic cigarette use is lower in Australia, compared to the United Kingdom, however the prevalence is increasing [27,28]. In 2016, an estimated 240,000 people reported using electronic cigarettes in Australia [29], and one of the primary reasons for interest or use in electronic cigarettes was to quit tobacco smoking [29,30]. With this increased popularity in the use of electronic cigarettes amongst people who smoke in Australia, it is important to examine how clinicians perceive the role of electronic cigarettes for smoking cessation, and what they say to patients when asked about the risks and benefits of electronic cigarettes.

Australia has adopted a precautionary approach to electronic cigarettes, and the regulatory framework is more restrictive than other countries such as the United Kingdom, Canada and New Zealand [27,28,31]. In Australia, non-nicotine containing electronic cigarettes can be bought legally. The purchase of nicotine for use as an e-liquid in Australia is illegal; however, it can be imported with a medical prescription for up to three months of personal therapeutic use [32,33]. This precautionary approach towards electronic cigarettes has been echoed in policy documents and recommendations from medical and health authorities in Australia citing concerns about the unknown health risks of electronic cigarettes for the general population [34,35,36,37]. However, this approach may place limitations on the use of electronic cigarettes in specific clinical populations and scenarios, such as around the time of surgery to reduce the perioperative harm caused by tobacco smoking [38,39,40].

Consistent, face-to-face smoking cessation advice and support from a multidisciplinary team of clinicians can engage patients in a quit attempt [41,42], to help them abstain from tobacco in the perioperative period and reduce their surgical risk. In the area of cardiothoracic surgery, the views and practices of anaesthetists [15] and thoracic surgeons [13,16] in the United States, the United Kingdom and Korea have been investigated to better understand how electronic cigarettes are viewed in the context of smoking cessation and the content of their patient discussions. These studies found that while most clinicians had engaged with their patients on the topic of electronic cigarettes, clinicians’ views about their safety and efficacy as a smoking cessation aid varied. Some clinicians had concerns about electronic cigarettes, and either did not recommend or discouraged their use [15,16]. Others believed the devices would help patients reduce or eliminate smoking, and either tolerated or recommended their use [13]. However, in Australia, the clinical role of electronic cigarettes as a smoking cessation aid is yet to be thoroughly explored, particularly through the perspectives of cardiothoracic clinicians, who have a crucial role in patient education, smoking cessation advice and support. To the best of our knowledge, the views and practices of clinicians from diverse disciplines, such as surgeons, anaesthetists, nurses and physiotherapists, in the cardiothoracic perioperative period in Australia have not been explored.

## 2. Methods

### 2.1. Design and Data Collection

This study was part of a larger study on smoking cessation care provided by cardiothoracic clinicians [8]. The research design included one-on-one, in-depth interviews with cardiothoracic surgeons, anaesthetists, nurses and physiotherapists in six hospitals in Sydney, NSW. These hospitals were responsible for approximately 43% of cardiothoracic cases in 2016 in NSW, with patients from urban, rural and remote areas, maximising potential generalisability [43]. Purposive sampling followed by a snowball sampling technique was used to identify the multidisciplinary clinicians involved in adult cardiothoracic surgery at three public tertiary referral hospitals and three private hospitals in Sydney, where the surgeons were also affiliated.

A formal invitation letter, participant information and consent form were sent (by email) to the head of cardiothoracic surgery at each hospital site, who subsequently identified other cardiothoracic surgeons at the six hospitals, and the heads of cardiothoracic anaesthetic, nursing and physiotherapy. The heads of these departments then nominated other appropriate staff involved in perioperative cardiothoracic care in their hospital. Multi-centre ethics approval was obtained from the Northern Sydney Local Health District Human Research Ethics Committee (LNR/15/HAWKE/356) and each clinician provided informed consent.

**Figure 1:**
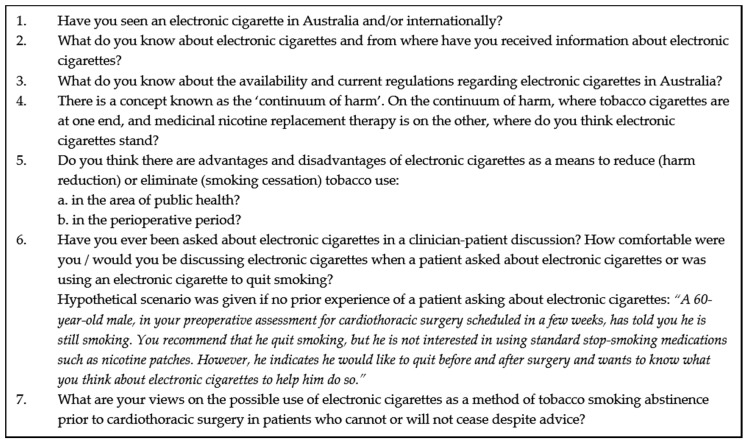
Interview Guide Questions.

A semi-structured, in-depth interview guide (Figure 1) was created, based on a review of the literature of electronic cigarette use in the area of coronary artery disease, lung cancer and cardiothoracic surgery. The interview guide also contained specific discussion topics, based on a previous U.S. study (with author’s permission) [15], to explore clinicians’ perceptions of electronic cigarettes as an aid to tobacco cessation in the perioperative period of cardiothoracic surgery. The interviews were conducted between October 2015 and November 2016 by one of the authors (NAL), a specialist physiotherapist in the area of cardiothoracic surgery, with 20 years of clinical experience. The mean interview time was 23 min (range 12 to 35). Information about the NSW Health guidelines on the 5A’s smoking cessation approach [44] were given to each clinician, if requested, at the end of each interview. The confidentiality and anonymity of participants was maintained at all times [45].

### 2.2. Data Analysis

Interviews were audio-recorded, professionally transcribed and de-identified. Each clinician was assigned a code based on their specialty-surgeons (S), anaesthetists (A), nurses (N) and physiotherapists (P). All data were imported to and managed in NVivo software version 11 [46]. Thematic analysis, as described by Braun and Clarke [47], was chosen as the best data analysis method to identify reoccurring patterns and themes from textual data derived from transcripts of the semi-structured qualitative interviews. As a study examining the views of a cohort from the same population (in this case, cardiothoracic clinicians), this data analysis approach is best able to systematically collate and group relevant responses from a variety of participants in relation to the study question [8,48].

An inductive approach was used, where Nia A. Luxton developed descriptive codes based on patterns observed in the data and conducted a critical analysis of these codes to collate them into major themes. The transcripts were also read by a co-author (Patti Shih) with extensive experience in qualitative methodology and use of NVivo, who developed themes independently. In addition, another member of the research team (Ross MacKenzie) (conducted double-coding of a subset of data in NVivo to ensure the final coding scheme had reliability. There was good agreement about the themes and any discrepancies were discussed among the three researchers until a consensus was reached. Supporting quotations were selected that generally expressed dominant views and demonstrated significant issues but also that reflected ‘deviant’ or ‘negative’ views [49]. As recommended when undertaking qualitative analysis [47], the process of analysis was recursive and often involved multiple iterations, particularly when identifying and refining themes from codes and categories.

## 3. Results

Fifty-two clinicians participated in the study: 15 cardiothoracic surgeons, 15 anaesthetists, 11 nurses, and 11 physiotherapists (Table 1). Their experience varied from recently qualified consultant anaesthetists and newly-graduated physiotherapists, to surgeons, anaesthetists, nurses and physiotherapists with more than 20 years of experience.

The analysis resulted in four themes (Table 2) on clinicians’ views towards electronic cigarettes and smoking cessation in the context of cardiothoracic surgery: (1) Electronic cigarettes were unlikely to be safe, but still safer than tobacco cigarettes; (2) Electronic cigarettes may have a harm reduction role in the context of public health; (3) Electronic cigarettes were a potential smoking cessation tool for the extraordinary circumstances of surgery; and (4) Patient-clinician discussions were influenced by clinician views about electronic cigarettes and clinicians’ discipline-specific professional role. These four themes were not mutually exclusive, but nonetheless represent distinct patterns in the transcripts.

### 3.1. Electronic Cigarettes Were Unlikely to Be Safe but Still Safer than Tobacco Cigarettes

While clinicians were aware of electronic cigarettes, they had limited knowledge of electronic cigarettes, how they worked, where they were made, and current regulations in Australia. The primary source of knowledge of clinicians was news and documentaries on popular media, such as radio and television, with many clinicians recounting media discussions of either tobacco industry involvement or uncertainty about the long-term harm caused by electronic cigarette use:

*“What have I picked up? From a medical point of view? Nowhere. This is from a media point of view—it’s a nicotine replacement, so it deals with cravings.”* (S12)

No clinician considered electronic cigarettes to be completely safe, and there were gradients in their views of the harm they would cause. The few clinicians who considered them to be unsafe also felt a complete ban on electronic cigarettes was appropriate. These clinicians considered that the physiological damage to the cardiac or respiratory system by electronic cigarettes would only show after years of use, similar to that of tobacco smoking.

*“It’s going to take years to get a handle on whether they are better or worse. Maybe it will cause other things we don’t even know about.”* (S1)

Other clinicians considered that electronic cigarettes were most likely safer than tobacco smoking but should be regulated in some manner until more scientific data was available. No clinician knew of the current Australian regulations on the sale or personal use of electronic cigarettes. There were a few clinicians who felt that, despite the uncertainty, electronic cigarettes were a viable form of nicotine replacement therapy (NRT) to help deal with nicotine cravings and should be available over the counter to encourage or enhance a quit attempt.

*“You can get NRT on a prescription, and over the counter. That could work for electronic cigarettes too.”* (A6)

### 3.2. Electronic Cigarettes May Have a Harm Reduction Role in the Context of Public Health

All clinicians viewed electronic cigarettes as the lesser of two evils compared to tobacco smoking.

*“Electronic cigarettes are nicotine and flavour. Even if the flavour is poisonous, it’s probably better than 3000 other chemicals in a tobacco cigarette.”* (A5)

On the continuum of harm with tobacco smoking at one end and NRT was at the other, the clinicians were divided on where electronic cigarettes sat. Those who regarded electronic cigarettes closer to tobacco in risk regarded electronic cigarettes either as a vehicle of the tobacco industry or a tool that hindered cessation, due to the similar mannerisms associated with smoking. This view was predominant among clinicians who were ex-smokers or had family members who currently smoked.

*“Electronic cigarettes still promote the oral component, so it would be too easy to slip back to smoking. And their use suggests that it’s still socially acceptable to put them in your mouth, renormalising smoking again.”* (S11)

Others felt electronic cigarettes sat further towards NRT as a cessation tool, as the hand-to-mouth component of electronic cigarettes was an advantage that filled the ‘space’ left by quitting tobacco cigarettes that NRT patches or gum did not fill.

*“I think people want to stop smoking. There’s those who can do it cold turkey or with nicotine replacement, but some need the hand-to-mouth kind of behaviour to continue. Whichever gets them off the cigarette.”* (S6)

The overall consensus was that electronic cigarettes may have a harm reduction role in the context of public health providing a person quit tobacco completely while using an electronic cigarette. However, due to the negative media messages and a likely tobacco industry involvement, electronic cigarettes were not regarded as a viable cessation tool for broader population tobacco control.

### 3.3. Electronic Cigarettes Were a Potential Smoking Cessation Tool for the Extraordinary Circumstances of Surgery

Electronic cigarettes were viewed by clinicians as a means of harm reduction in the truest sense of the words. All clinicians expressed the desire for a patient to abstain from tobacco prior to surgery for as long as possible to reduce the known harm caused by continued tobacco smoking in the perioperative period of cardiothoracic surgery. Nevertheless, some questioned the need for electronic cigarettes, citing experience of patients successfully quitting with NRT, as a result of the enforced abstinence once hospitalised for cardiothoracic surgery, or quitting ‘cold turkey’ (abrupt abstinence).

*“I see so many patients who they stop from 60 a day to nil just by not relenting. That’s probably part of the reason, maybe the motivation and the mental attitude to that. So, if they want to do it, they can do it without the electronic cigarette.”* (S15)

Clinicians had discipline-specific views about the role of electronic cigarettes as an appropriate cessation method in the perioperative period. Surgeon, anaesthetists and nurses who specialised more in the care of coronary artery bypass surgery patients were opposed to nicotine use in any form, due to the risk of perioperative coronary artery vasoconstriction and tachycardia. For those surgeons, nurses and anaesthetists, abrupt cessation was the preferred method of quitting prior to surgery with continued abstinence postoperatively. Similarly, surgeons, anaesthetists and physiotherapists who specialised more in the care of thoracic surgery patients, or who had first-hand experience of the negative impact of tobacco smoking on postoperative pulmonary complications, were concerned about the adverse effects of the inhaled aerosols from electronic cigarettes, and the risk of bronchospasm or airway harm.

*“I have concerns about the flavours, because you don’t know what’s in it. They’re inhaling a whole cocktail of things before the anaesthetic.”* (A4)

Most clinicians, however, felt that completely switching to electronic cigarettes to achieve tobacco abstinence prior to surgery would reduce the known pathophysiologic consequences of continued tobacco smoking on a patient’s surgical outcomes. These clinicians acknowledged that electronic cigarettes could provide a bridge between tobacco smoking and NRT use, a pathway to cessation of all cigarettes, or a novel method for patients who required nicotine delivery in a different manner.

*“If using electronic cigarettes was a way of getting higher concentrations of nicotine as a single hit, which some people seem to need, that would be worthwhile, because other means of nicotine therapy are delivered too slow, compared to tobacco cigarettes.”* (A12)

All clinicians had numerous examples of patients who had been unable, or unwilling, to quit tobacco smoking prior to surgery, or who had resumed smoking after surgery, including: patients who had experienced severe adverse effects of pharmacotherapy, such as varenicline, or nicotine withdrawal, and did not want to try to quit again; patients who relied on smoking as a method to manage stress; patients who had little confidence in their ability to quit; and patients with complex sociodemographic situations or mental illness. Most clinicians, even those with negative views about the safety of electronic cigarettes, conceded that they may have a role to play to create a quit attempt prior to surgery.

*“I think there are patients who are so habituated to smoking that if electronic cigarette use is the only way they can stop, I accept that.”* (S10)

However, no clinician condoned the use of electronic cigarettes postoperatively. The enforced abstinence from smoking in the hospital smoke-free environment and the acuity of a patient’s illness and surgery was seen as a teachable moment for patients to quit all forms of cigarettes.

### 3.4. Patient-Clinician Discussions Were Influenced by Clinician Views about Electronic Cigarettes and Clinicians’ Professional Role

No clinician had been asked about electronic cigarettes by a patient awaiting cardiothoracic surgery at the time of this study. Therefore, all clinicians were given a hypothetical scenario (Figure 1, question 6) to explore what they would say if a patient asked about electronic cigarettes to abstain from smoking in the perioperative period. Anaesthetists and physiotherapists who regarded electronic cigarettes as a risk to a patient’s respiratory system said they would reiterate the uncertainties about electronic cigarettes compared to other methods and recommend that the patient discuss their use with their surgeon.

*“I would tell the patient that it’s good that they’re showing steps to try and stop smoking, but they would need to talk to their surgeon about electronic cigarettes. They’re not harmless.”* (P5)

Other anaesthetists and nurses who had a direct line of contact with the surgeon in their role, would seek the surgeon’s advice.

*“I would talk to the surgeons and ask what their opinion was. I would have to get more information because I wouldn’t want to recommend something that I know nothing about.”* (N9)

Surgeons who regarded electronic cigarettes negatively, as an unknown and most likely harmful entity, said they would be unequivocal in their advice to patients, highlighting the risks and unproven efficacy of electronic cigarettes as a smoking cessation tool. Surgeons who regarded electronic cigarettes more positively said they would highlight the current uncertainty about electronic cigarettes’ safety but would recommend a patient quit tobacco before surgery using any means.

*“You have to use whatever means are appropriate to protect the patient from themselves and to optimise their surgical outcome in the short term and their life outcome in the long term.”* (S14)

Anaesthetists, nurses and physiotherapists, while uncertain about the risks and benefits of electronic cigarettes, felt they would use the patient’s question about electronic cigarettes and guide it to a broader conversation about quitting tobacco use before surgery. Some would guide patients away from electronic cigarettes to evidence-based cessation, such as NRT or Quitline (an Australian telephone services that provides smoking cessation information, advice, and support). Others felt comfortable recommending the patient try electronic cigarettes, as the attempt may achieve the intended tobacco abstinence prior to surgery.

*“I would be comfortable recommending an electronic cigarette because I think it achieves the outcome that we want for the patient.”* (A14)

## 4. Discussion

This study provides the first in-depth views of Australian clinicians towards electronic cigarettes in the perioperative period of cardiothoracic surgery. It shows that a number of Australian clinicians see a role for electronic cigarettes in a specific clinical setting to achieve tobacco abstinence prior to surgery, for patients who have been unable to quit with other cessation methods. The study reinforces international findings among clinicians involved in the care of patients with tobacco-related diseases: clinicians have a lack of knowledge and familiarity about electronic cigarettes; media is a primary source of clinicians’ information; and clinicians perceive that electronic cigarettes were likely to have some adverse effects [15,16]. Whilst there were variations in views among professions, the consensus was that, compared to known physiological harm of combustible tobacco cigarettes, electronic cigarettes were less harmful [13,15].

In Australia, the primary cause of coronary artery disease and lung cancer is tobacco smoking [50], and the need for cardiothoracic surgical management of these diseases will continue. Clinicians are trusted sources of information and their advice has been found to create higher levels of tobacco quit attempts and cessation success [51,52,53], particularly among patients who are at their most vulnerable in the perioperative period [54]. Because of the current uncertainty about the safety of electronic cigarettes, and the influence that a clinician’s own beliefs about smoking cessation methods can have [8], this study shows that patients may be given mixed messages about electronic cigarettes. For example, certain surgeons, anaesthetists and nurses neither endorsed NRT or electronic cigarette use in the perioperative period of coronary artery bypass surgery, due to the potential adverse effects of nicotine on diseased and newly grafted coronary arteries, whilst others were comfortable recommending any method that would lead to tobacco abstinence. Therefore, the information and acceptance of smoking cessation methods may differ depending on the clinician’s views, their profession, and the hospital they attend.

Previous international surveys and interview studies of clinicians in areas related to tobacco-induced diseases have reported both optimism and scepticism about the benefits of electronic cigarettes as a tool to reduce or cease tobacco cigarettes, which influenced the content of their conversations with patients [13,15,16,17]. The range of views found in these studies were echoed in this Australian study, with clinicians either discouraging, tolerating or encouraging electronic cigarettes, based on uncertainty and concern about electronic cigarettes at one end to the view that whatever helped a patient quit tobacco smoking before surgery was worth considering. This diversity of opinions from such a variety of international and Australian professions—physicians, oncologists, surgeons, nurses and physiotherapists—emphasises the need to provide education and guidance to all clinicians, in order to create consistency in the advice offered to patients, irrespective of a country’s regulation or a clinician’s personal opinions about electronic cigarettes. Apart from the recent changes in the United Kingdom [26], there is little translation of the extensive position papers and recommendations from professional clinical societies, governments and health authorities to formally guide the patient-clinician discussion about the use of electronic cigarettes to quit smoking [13,15]. Until scientific evidence on the safety and efficacy of electronic cigarettes resolves the current debate, clear and accessible guidance detailing the risks, benefits and uncertainties of using electronic cigarettes should be provided to clinicians who care for cardiothoracic surgical patients. This could take the form of written literature in the hospital pre-admission clinics, surgeons’ rooms, or cardiothoracic surgical wards, that clinicians can refer to, or decision aids designed to facilitate clinician-patient discussions regarding tobacco use around the time of surgery [55].

The provision of smoking cessation care to patients throughout the perioperative period is a recognised goal shared by clinicians responsible for the surgical management of patients [7,56,57,58]. This novel study reports the views of typical Australian interdisciplinary teams of clinicians—surgeons, anaesthetists, nurses and physiotherapists—about a relatively novel consumer product, in a country where electronic cigarettes are tightly regulated. Indeed, no surgeon, anaesthetist, nurse or physiotherapist in the study had experienced a patient-clinician discussion about electronic cigarettes, which differs from the other international studies [13,15,16]. In this study, the clinicians’ positive views towards electronic cigarettes as a short-term alternative to tobacco cigarettes prior to surgery was primarily based on both their clinical experience and knowledge of the definite harm caused by continued tobacco smoking on patient outcomes, and their recognition of the difficulties some patients faced in quitting despite imminent cardiothoracic surgery. The view that electronic cigarettes could be used to achieve tobacco abstinence for patients who were not successful in prior quit attempts with approved therapies is consistent with the views of numerous clinicians who care for patients with tobacco-related diseases [12,13,14,15,17,18].

The results of this study highlight an absence of real-world clinician experience responding to patients’ questions about electronic cigarettes, compared with studies in the United States, United Kingdom, Greece and Korea [12,13,14,15,16,17,18]. The lack of surgical patient-clinician discussions on electronic cigarettes at the time of this study (October 2015 to November 2016) suggest that the regulations in Australia had the desired effects, with less use and interest in electronic cigarettes compared to other countries with fewer regulations and higher use [27,28,59]. Additionally, it may also reflect the characteristics of certain patients who actively smoke, despite their diagnosis and imminent surgery; such characteristics include a high nicotine dependence, a reliance of smoking to reduce anxiety, or a lack of awareness of the immediate risks of tobacco use in the perioperative period [7,60]. While people continue to smoke tobacco, the need for cardiothoracic surgical management will also continue as the population ages and lives longer. As the prevalence of electronic cigarette use is increasing in Australia [29,30], Australian interdisciplinary clinicians may find themselves increasingly involved in discussions about electronic cigarettes, with patients who have tried other methods to quit and are interested in the use of electronic cigarettes in a subsequent quit attempt [61].

There are both strengths and limitations to this study. Firstly, this study draws from a specific sample of clinicians from metropolitan hospitals in Sydney, Australia, who were responsible for 43% of cardiothoracic surgery cases in NSW in 2016 [62]. Therefore, the findings are context specific to the settings and selected individuals involved. While this means that the results cannot be directly generalised to other settings in Australia or internationally, the variety of disciplines and experience of the clinicians interviewed has produced an in-depth and extensive understanding of the perspectives and approaches that arise in their clinician-patient discussion of smoking cessation in cardiothoracic surgical contexts. These findings can therefore provide a point of comparison and contrast for other studies examining similar issues, albeit in different geographic and cultural settings. Secondly, the method of participant recruitment for the anaesthetists, nurses and physiotherapists could lead to selection bias. Yet, this is the most ethically and logistically appropriate approach as the department head is the best person to know the workload of each clinician and their willingness to carve out extra time for study participation. Having said this, at each study site there are a limited number of specialist clinicians involved in the interdisciplinary perioperative care of cardiothoracic patients. By the end of the study, only 10% of the clinicians were not recruited. This, therefore, controlled for some of this possible participant bias. Thirdly, this study did not explore the clinicians’ views regarding the efficacy of non-nicotine containing electronic cigarettes as a smoking cessation tool, or their willingness to write a prescription for nicotine e-liquid. These questions could be included in future research of other Australian clinical professions who care for patients with tobacco-related diseases, such as in the areas of oncology or respiratory medicine. Finally, since the interviews were conducted however, there has been an Australian parliamentary inquiry [63] into electronic cigarettes, with submissions by numerous medical and health authorities, and much media about electronic cigarettes. Whilst no changes have been made to current regulations, the intense media discussions may have altered some of the clinicians’ views. Nevertheless, these qualitative findings add to previous international quantitative research in cardiothoracic surgery [13,15,16], confirming that the lack of scientific evidence of the safety and efficacy of electronic cigarettes as a smoking cessation aid has an impact on the views of clinicians and patient-clinician discussions.

## 5. Conclusions

This study represents the only known study of the views and practices of interdisciplinary Australian clinicians involved in the care of cardiothoracic surgical patients, and adds a new perspective to previous surveys of international clinicians, as it is an in-depth, qualitative study in Australia, where the regulatory framework is complex and unique. Whilst the findings of the study reveal the limited knowledge about electronic cigarettes and their uncertainty about the long-term safety of electronic cigarettes, it adds to the evidence regarding positive attitudes of clinicians who care for patients with tobacco-related diseases. In the extraordinary context of the perioperative period, where continued tobacco smoking is known to cause an increase in surgical risk, electronic cigarettes may engage patients in a quit attempt that can be guided and supported with a common aim towards long-term smoking cessation. As the debate about electronic cigarette continues, played out in the media, clinicians are likely to be receiving more frequent questions from patients about electronic cigarettes as a cessation aid. This reinforces the need for clearer and balanced guidelines for Australian clinicians on the topic of electronic cigarettes and cardiothoracic surgery.

## Figures and Tables

**Table 1:** Characteristics of the participating clinicians (*n* = 52).

Characteristic	Surgeons	Anaesthetists	Nurses	Physiotherapists
Gender (male), *n* (%)	15 (100%)	13 (87%)	1 (9%)	1 (9%)
Age (year), *n* (%)				
<40				5 (45%)
>40	15 (100%)	15 (100%)	11 (100%)	6 (55%)
Current work setting, *n* (%)				
Public hospital	15 (100%)	15 (100%)	8 (73%)	5 (45%)
Self-reported time working in cardiothoracic surgical area (year), *n* (%)				
<10	2 (13%)	11 (73%)	4 (36%)	7 (64%)
>10	13 (87%)	4 (27%)	7 (64%)	4 (36%)

**Table 2:** Themes relevant to clinicians’ views of electronic cigarettes.

Theme	Sub Theme	Categories	Professions and Frequency			
			Anaesthetists (*n* = 15)	Surgeons (*n* = 15)	Nurses (*n* = 11)	Physiotherapists (*n* = 11)
Electronic cigarettes were unlikely to be safe but still safer than tobacco cigarettes	Limited knowledge of electronic cigarettes	Media was the main source of information	15	15	11	11
		Electronic cigarettes should be banned or regulated until further evidence available	2	5	3	0
		Unsure of how electronic cigarettes should be regulated due to lack of evidence	10	9	8	11
		Electronic cigarettes should be available over-the-counter/tobacconist	2	1	0	0
Electronic cigarettes may have a harm reduction role in the context of public health	Positive views of electronic cigarettes	Electronic cigarettes as the lesser of two evils	8	7	4	6
		Hand to mouth similarities as an alternative form of nicotine replacement therapy (NRT)	4	5	2	2
	Negative views of electronic cigarettes	Electronic cigarettes were too similar to tobacco cigarettes	3	3	5	3
Electronic cigarettes were a potential smoking cessation tool for the extraordinary circumstances of surgery	Electronic cigarettes as an alternative to tobacco smoking	If patients had tried other methods and were unable to quit	5	4	3	4
		As a bridge off tobacco smoking before surgery	4	6	2	2
	Clinicians’ preferred methods outweighed potential role of electronic cigarettes	Preference for evidence-based methods of NRT	2	2	5	1
		No nicotine in any form allowed for their patients prior to surgery	1	2	1	0
		Unknown effects of vaping on patients’ airways	3	1	0	4
Patient-clinician discussions were influenced by clinician views about electronic cigarettes and clinicians’ professional role	Consider patient short-term use of electronic cigarettes before surgery	Comfortable with discussing electronic cigarette short-term patient use to help stop tobacco smoking prior to surgery	8	7	1	2
	Discourage patient use of electronic cigarettes	Comfortable with discussing the lack of evidence being their reason for not recommending electronic cigarettes	1	4	2	1
	Unsure and would seek advice	Emphasis on patient’s choice to use electronic cigarettes due to lack of own knowledge	6	4	8	8
